# Gromwell ameliorates glucocorticoid-induced muscle atrophy through the regulation of Akt/mTOR pathway

**DOI:** 10.1186/s13020-024-00890-5

**Published:** 2024-01-29

**Authors:** Ahyoung Yoo, Jung-In Kim, Hyunjung Lee, Farida S. Nirmala, Jeong-Hoon Hahm, Hyo Deok Seo, Chang Hwa Jung, Tae Youl Ha, Jiyun Ahn

**Affiliations:** 1 Aging and Metabolism Research Group, Korea Food Research Institute, Wanju-gun, 55365 Korea; 2 Division of Food Biotechnology, University of Science and Technology, Daejeon, 34113 Korea

**Keywords:** Gromwell, *Lithospermum erythrorhizon*, Dexamethasone, Muscle atrophy, Lithospermic acid, mTOR/glucocorticoid receptor

## Abstract

**Background:**

Muscle atrophy is characterized by decreased muscle mass, function, and strength. Synthetic glucocorticoids, including dexamethasone (Dexa), are commonly used to treat autoimmune diseases. However, prolonged exposure of Dexa with high dose exerts severe side effects, including muscle atrophy. The purpose of this study was to investigate whether Gromwell root extract (GW) can prevent Dexa-induced muscle atrophy in C2C12 cells and mice and to characterize the composition of GW to identify bioactive compounds.

**Methods:**

For in vitro experiments, GW (0.5 and 1 µg/mL) or lithospermic acid (LA, 5 and 10 µM) was added to C2C12 myotubes on day 4 of differentiation and incubated for 24 h, along with 50 µM Dexa. For in vivo experiment, four-week-old male C57BL/6 mice were randomly divided into the four following groups (*n* = 7/group): Con group, Dexa group, GW0.1 group, and GW0.2 group. Mice were fed experimental diets of AIN-93 M with or without 0.1 or 0.2% GW for 4 weeks. Subsequently, muscle atrophy was induced by administering an intraperitoneal injection of Dexa at a dose of 15 mg/kg/day for 38 days, in conjunction with dietary intake.

**Results:**

In Dexa-induced myotube atrophy, treatment with GW increased myotube diameter, reduced the expression of muscle atrophy markers, and enhanced the expression of myosin heavy chain (MHC) isoforms in C2C12 cells. Supplementation with the GW improved muscle function and performance in mice with Dexa-induced muscle atrophy, evidenced in the grip strength and running tests. The GW group showed increased lean body mass, skeletal muscle mass, size, and myosin heavy chain isoform expression, along with reduced skeletal muscle atrophy markers in Dexa-injected mice. Supplementation with GW increased protein synthesis and decreased protein degradation through the Akt/mammalian target of rapamycin and glucocorticoid receptor/forkhead box O3 signaling pathways, respectively. We identified LA as a potential bioactive component of the GW. LA treatment increased myotube diameter and decreased the expression of muscle atrophy markers in Dexa-induced C2C12 cells.

**Conclusions:**

These findings underscore the potential of the GW in preventing Dexa-induced skeletal muscle atrophy and highlight the contribution of LA to its effects.

**Supplementary Information:**

The online version contains supplementary material available at 10.1186/s13020-024-00890-5.

## Introduction

Muscles constitute more than 40% of the total body weight of the human body [1] and play important physiological roles in movement, energy storage, and metabolism [2, 3]. Muscle atrophy is defined as a decrease in muscle mass, function, and strength [4]. It reduces the quality of life due to increased risks of frequent falls and decreased walking ability. The occurrence of muscle atrophy is on the rise due to demographic aging and extended life expectancy [5]. This is caused by diseases and conditions such as glucocorticoid excess, cachexia, denervation, disuse, or malnutrition [6]. The mechanisms underlying the development of muscle atrophy are not fully understood but are considered to be related to increased proteolysis due to a decrease in muscle protein synthesis [7], increased ubiquitin-proteasome system (UPS) activity [8], increased reactive oxygen species production [9], increased inflammation [10], and mitochondrial dysfunction [11]. Currently, no effective drugs or treatments for muscle atrophy are available.

Synthetic glucocorticoids such as dexamethasone (Dexa) are commonly used to treat various autoimmune diseases including rheumatoid arthritis, systemic lupus erythematosus, and bronchial asthma [12]. However, the prolonged and high dosage of Dexa may lead to side effects, notably muscle atrophy [13]. When Dexa binds to the glucocorticoid receptor (GR) and is translocated to the nucleus, it increases UPS activity, promoting the expression of E3 ligases such as Atrogin-1 and Muscle RING-Finger protein 1 (MuRF1), leading to the ubiquitination of muscle proteins [8]. The regulation of E3 ligases involves transcription factors such as forkhead box O3 (FoxO3), which, in turn, is negatively modulated by Akt signaling [14]. Insulin-like growth factor, upstream of the mammalian target of rapamycin (mTOR) in the Akt/mTOR signaling pathway, has been well characterized for its role in muscle growth through the regulation of mTOR phosphorylation [15]. It induces skeletal muscle protein synthesis by altering the phosphorylation of its downstream receptors, ribosomal protein S6 kinase 1 (S6K1) and eukaryotic translation initiation factor 4E-binding protein 1 (4EBP1) [16]. Dexa decreases muscle protein synthesis by downregulating the Akt/mTOR signaling pathway [17]. Therefore, finding potential substances that regulate protein degradation and synthesis signaling pathways may be a strategy to prevent muscle atrophy [18].

Gromwell (*Lithospermum erythrorhizon*) is a perennial herbaceous plant belonging to the Boraginaceae family, native to East Asia, particularly Japan, Korea, and East China [19]. In China, it has been used as a folk remedy for thousands of years to treat burns, sore throats, macular eruption, measles, and carbuncles [20]. The Gromwell root extract (GW) exerts antibacterial [21], antitumor [22], and antiangiogenic [23] effects. We previously reported that GW suppresses high-fat diet-induced obesity in mice [24]. However, the effects of GW on muscle atrophy have not yet been reported.

In this study, we investigated whether the GW could prevent Dexa-induced muscle atrophy in C2C12 cells and mice. We also elucidated the composition of the GW, with emphasis on identifying the bioactive compound.

## Materials and methods

### Preparation of GW

Dried GW roots were ground and extracted twice. The first extraction was performed with 50% ethanol at 30 ℃ for 8 h with shaking, and the second was performed with 50% ethanol at 30 ℃ for 6 h with shaking. The extract was filtered and concentrated to a brix of 20–30 at a temperature below 60℃. After mixing 20% dextrin relative to the extracted content, it was sterilized at 90 ℃ for 30 min and freeze dried.

### High-performance liquid chromatography (HPLC) analysis

HPLC analysis for lithospermic acid (LA; PHL80491, purity: 90%, Sigma-Aldrich, St. Louis, MO, USA) was performed using an Agilent 1200 series HPLC system (Agilent Technologies, CA, USA) with C18 columns (250 × 4.6 mm I.D. 5 μm, YMC, Kyoto, Japan). Absorbance was measured at 312 nm using a diode array detector (Agilent Technologies). Solvent A, which comprised 0.1% formic acid in H_2_O, and Solvent B, acetonitrile, were used for HPLC analysis. The gradient program began with an isocratic A/B ratio of 80:20 for the first 5 min and then transitioned to an A/B ratio of 75:25, which was maintained for 5–30 min. Following this phase, the gradient shifted to an A/B ratio of 80:20 in the 30–35 min interval and concluded with an isocratic A/B ratio of 80:20 from 35 to 40 min. The column temperature was maintained at 25 °C. HPLC analysis for shikonin (S7576, purity: 98%, Sigma-Aldrich) was performed using JASCO liquid chromatography (JASCO, Tokyo, Japan) with C18 columns (250 × 4.6 mm I.D. 5 μm, YMC). Absorbance was measured at 516 nm using a JASCO PDA detector. Solvent A, comprising 0.1% acetic acid in H_2_O, and Solvent B, i.e., acetonitrile, were used for HPLC analysis. The gradient program was initiated with an isocratic A/B ratio of 85:15 for the first 2 min and then transitioned to an A/B ratio of 70:30, which was maintained subsequently for 2–12 min. Following this phase, the gradient shifted to an A/B ratio of 35:65 in the 12–15 min interval and was ultimately concluded with an isocratic A/B ratio of 20:80 from 15 to 25 min. The column temperature was maintained at 40 °C. The flow rate and injection volume were set at 1 mL/min and 10 µL for both conditions, respectively.

### C2C12 cell culture and differentiation

Murine myoblast C2C12 cells (ATCC, Manassas, VA, USA) were maintained in Dulbecco’s Modified Eagle Medium with 4500 mg/L high-glucose (DMEM; Hyclone Co., Logan, UT, USA) containing 10% fetal bovine serum (FBS; Hyclone) and 1% penicillin-streptomycin (Gibco, Invitrogen Inc., Carlsbad, CA, USA) solution. The cells were grown in an incubator at 37 °C with 5% CO_2_. For differentiation, 100% confluent cells were exposed to a differentiation medium containing high-glucose DMEM and 2% horse serum (Gibco, Invitrogen Inc.). To observe early differentiation markers, cells were treated with 2% horse serum medium containing LA (5 and 10 µM) for 2 d. To assess the effect on muscle atrophy, GW (0.5 and 1 µg/mL) or LA (5 and 10 µM) was added to differentiated myotubes on day 4 of differentiation and incubated for 24 h, along with 50 µM Dexa (D4902, Sigma-Aldrich).

### Immunofluorescence (IF) assay

The fixation of differentiated C2C12 myotubes occurred for 30 min in 4% formaldehyde, followed by permeabilization using 0.05% saponin in phosphate-buffered saline (PBS) and subsequent blocking with 1% bovine serum albumin in PBS. Staining of the cells involved the use of total myosin heavy chain (MHC) antibody (DSHB, Lowa City, IA, USA) or α-actinin antibody (Sigma-Aldrich), followed by application of an Alexa Fluor 488-conjugated mouse secondary antibody (Cell Signaling Biotechnology, Beverly, MA, USA) and 4ʹ,6-diamidino-2-phenylindole (Molecular Probes, OR, USA) in PBS. The stained cells were observed under a fluorescence microscope (Olympus, Tokyo, Japan), and the myotube diameters were analyzed using ImageJ software (NIH, Bethesda, MD, USA).

### Animal experiments

Three-week-old male C57BL/6 mice were obtained from Orient Bio, Inc. (Seongnam, Korea). All animal studies were approved by the Korea Food Research Institute (KFRI-IACUC, KFRI-M-21,064) and maintaining controlled conditions at 22 ± 3 °C, the mice followed a consistent 12 h light and 12 h dark cycle. After 1 week of acclimatization, 28 mice were randomly divided into the four following groups (*n* = 7/group): group I, the vehicle-control group (Con group); group II, the Dexa-injected group (Dexa group); and group III, the Dexa-injected with 0.1% GW-supplemented group [100 mg/kg body weight (BW)]; and group IV, the Dexa-injected with 0.2% GW-supplemented group (200 mg/kg BW). Mice were fed experimental diets of AIN-93 M with or without 0.1 or 0.2% GW for 4 weeks. Subsequently, muscle atrophy was induced by administering an intraperitoneal injection of Dexa at a dose of 15 mg/kg/day for 38 d, in conjunction with dietary intake. Post-treatment, all mice were anesthetized with 2.5% isoflurane and euthanized to obtain muscle tissues.

### Measurement of muscle Mass and Performance

Dual-energy X-ray absorptiometry (DXA; InAlyzer, Medikors, Korea) was employed to determine the lean body mass and body fat mass for each mouse. Using a grip strength meter (Bioseb, Chaville, France), we measured the muscle grip strength by having the mice grasp the grid with their forelimbs, calculating the mean of five consecutive measurements for each animal. Following two days of training, we measured the total running distance and time using a rodent treadmill (Ugo Basile, Varese, Italy) set at a 10° incline. After adaptation, the running distance and time were measured. Starting at 10 m/min and lasting 10 min, the mice experienced incremental speed increments of 2 m/min every 3 min, ensuring the maximum speed did not surpass 20 m/min. The endpoint was set when the mice contacted the shock grid for 10 s.

### Histological analysis

To determine the cross-sectional area (CSA), we preserved the gastrocnemius (Gas) muscles in OCT-embedded blocks at −80 ℃. Sections measuring 7 μm in thickness were obtained from the tissue block using a low-temperature cryo-microtome (CM 1850, Leica Microsystems, Wetzlar, Germany) and placed onto glass slides. Tissue sections were fixed in ice-cold 20% acetone for 20 min and blocked with 10% FBS in PBS for 1 h. The tissues were incubated overnight at 4 ℃ with the anti-laminin antibody. The tissues were treated with Alexa Fluor 488 for 30 min. After staining, the tissues were washed twice with PBS and mounted using Fluoroshield (F6182, Sigma Aldrich). Images of stained tissue sections were acquired using an Axio Imager Z2 microscope (Carl Zeiss, Jena, Germany).

### RNA extraction and quantitative real-time PCR (qRT-PCR) analysis

Total RNA was extracted from animal muscle tissues using the RNeasy Mini Kit (Qiagen, Germantown, MD, USA) for cells and the RNeasy Fibrous Tissue Mini Kit (Qiagen). cDNA was synthesized using the ReverTra Ace qPCR RT Master Mix (Toyobo, Osaka, Japan). qRT-PCR was performed using SYBR Green Master Mix (Toyobo) on the ViiA7 RT-PCR System (Applied Biosystems, Foster City, CA, USA). Sequences of the primers used were Atrogin-1, F:5’- AAGGCTGTTGGAGCTGATAGCA − 3,’ R:5’- CACCCACATGTTAATGTTGCCC − 3’; MuRF1, F:5’- TGTCTCACGTGTGAGG TGCCTA − 3,’ R:5’- CACCAGCATGGAGATGCAGTTAC − 3’; myoblast determination protein 1 (MyoD), F:5’- CCGTGTTT CGACTCACCAGA − 3,’ R:5’- GTAGTAGGCGGTGTCGTAGC − 3’; myogenin (MyoG), F:5’-GTAGTAGGCGGTG TCGTAGC − 3,’ R:5’- CCACGATGGACGTAAGGGAG − 3’; 18s, F:5’- CTCAACA CGGGA AACCTCAC − 3,’ R:5’- CGCTCCACCAACTAAGAACG − 3’.

### Protein extraction and western blot analysis

Total protein isolation utilized the radioimmunoprecipitation assay lysis buffer (Thermo Fisher Scientific, Waltham, MA, USA), while the NE-PER™ Nuclear and Cytoplasmic Extraction Reagents kit (Thermo Fisher Scientific) was used to prepare nuclear and cytosolic fractions. A protein concentration of 20 µg was subjected to sodium dodecyl-sulfate polyacrylamide gel electrophoresis and separated proteins were transferred onto polyvinylidene fluoride membranes (Bio-Rad, Hercules, CA, USA). Blocking of the membranes occurred through the use of 5% skim milk in Tris-buffered saline and Tween 20 (TBST), followed by an overnight incubation at 4 °C with primary antibodies. After multiple washes with TBST, the membranes were exposed to the suitable horseradish peroxidase-conjugated secondary antibody. Protein bands were visualized using enhanced chemiluminescence reagent (Bio-Rad). The primary antibodies used are listed in Additional file 1: Table S1.

### Statistical analysis

Data are expressed as mean ± standard deviation (SD) for in vitro studies and as mean ± standard error of the mean (SEM) for in vivo studies. Data were subjected to statistical analysis using the GraphPad Prism 10 software (San Diego, CA, USA). One-way analysis of variance (ANOVA) was used to compare quantitative data among groups, followed by the Bonferroni post-hoc test. *p* < 0.05 indicated statistical significance.

## Results

### Effect of GW on Dexa-induced muscle atrophy in differentiated C2C12 cells

**Fig. 1:**
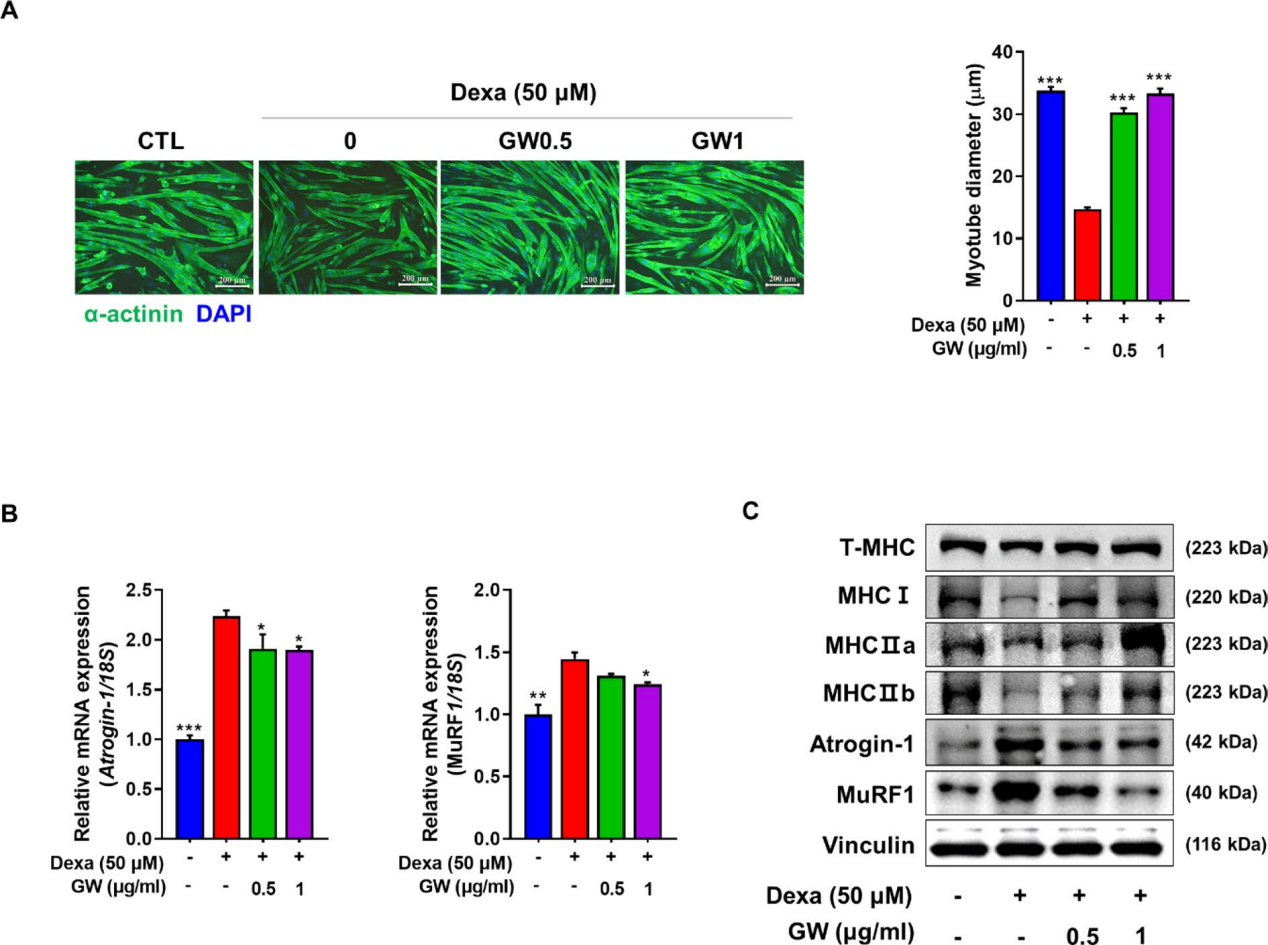
Effect of GW on Dexa-induced myotube atrophy. **A** Differentiated C2C12 cells were fixed and stained with α-actinin antibody (scale bar, 200 μm) (left). Myotube diameter was calculated as the average diameter of α-actinin-positive multinucleated myotubes (right). **B** The expression levels of Atrogin-1 and MuRF1 quantified by qRT-PCR in Dexa-treated myotubes. **C** The protein levels of total MHC, MHCI, IIa, IIb, Atrogin-1, MuRF1, and Vinculin measured by western blot in Dexa-treated C2C12 cells. Results are expressed as mean ± SD. One-way ANOVA was used to compare more than two groups, followed by Bonferroni post-hoc test. * p < 0.05, ** p < 0.01, *** p < 0.001 versus the Dexa-treated myotubes

To compare the efficacy of GW in preventing muscle atrophy under different ethanol extraction conditions, we performed qRT-PCR analysis to assess the expression of Atrogin-1 and MuRF1 genes. Both 50 and 80% ethanol extraction conditions reduced the expression of Atrogin-1 and MuRF1 compared to Dexa-induced myotubes. Because there was no difference between the extraction conditions, subsequent experiments used a 50% ethanol extract with lower ethanol intensity (Additional file 1: Fig. S1). C2C12 cells were treated with GW for 24 h and MTT was measured. We observed a survival rate of ≥ 90% for all tested concentrations (Additional file 1: Fig. S2A). To investigate the effects of the GW on Dexa-induced muscle atrophy, C2C12 myotubes were simultaneously treated with Dexa and GW for 24 h. IF staining of α-actinin confirmed that Dexa treatment induced myotube atrophy, causing a significant decrease in myotube diameter compared to the control, whereas GW restored it to the levels in the control. Myotube diameter decreased from 33.78 to 14.72 μm following Dexa treatment, whereas it increased to 30.29 μm post treatment with GW at 0.5 µg/mL and 33.32 μm with treatment at 1 µg/mL (Fig. 1A). We determined the effect of the GW on the mRNA expression of the muscle atrophy-related genes, Atrogin-1 and MuRF1. These atrogenes were downregulated following treatment with GW compared to those in the Dexa-only group (Fig. 1B). Western blot analysis demonstrated that the total MHC and MHC isomers such as MHCI, MHCIIa, and MHCIIb were decreased after Dexa treatment and increased following treatment with the GW. The protein expressions of Atrogin-1 and MuRF1 increased following Dexa treatment and decreased with GW treatment (Fig. 1C).

### Effect of GW on grip strength and muscle performance in Dexa-induced muscle atrophy

**Fig. 2:**
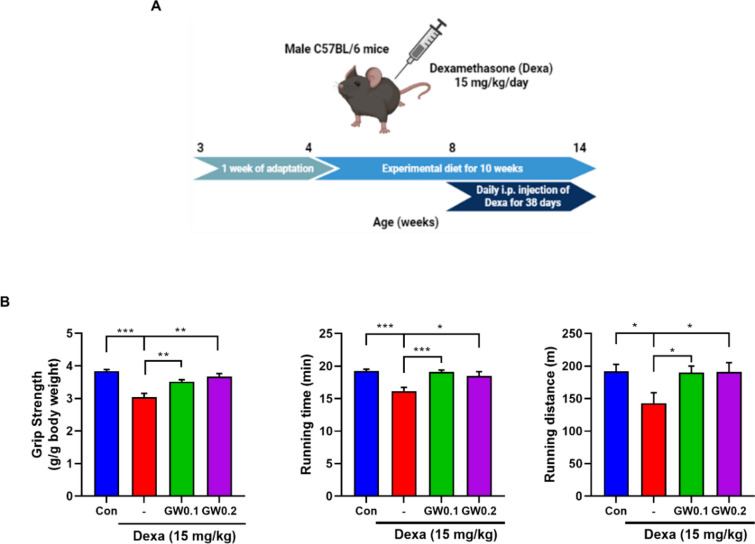
Effect of GW on grip strength and muscle performance in Dexa-induced muscle atrophy. **A** Experimental design. **B** Muscle function was measured on grip strength (g/g BW). Muscle performance was measured by total running time to exhaustion (min) and running distance (m). Results are expressed as mean ± SEM. One-way ANOVA was used to compare more than two groups, followed by Bonferroni post-hoc test. * p < 0.05, ** p < 0.01, *** p < 0.001 versus the Dexa group

Four-week-old C57BL/six male mice were fed the experimental diet for 10 weeks and injected with Dexa for the last 38 d to induce muscle atrophy (Fig. 2A). We examined the grip strength to measure muscle function. Compared to the Dexa group, grip strength was increased by 13.42% in the GW0.1 group and 17.06% in the GW0.2 group. Muscle performance was assessed on a treadmill, revealing significant increases in both running time and distance in the GW group compared to the Dexa group (Fig. 2B).

### Effect of GW on muscle mass in Dexa-induced muscle atrophy

**Fig. 3:**
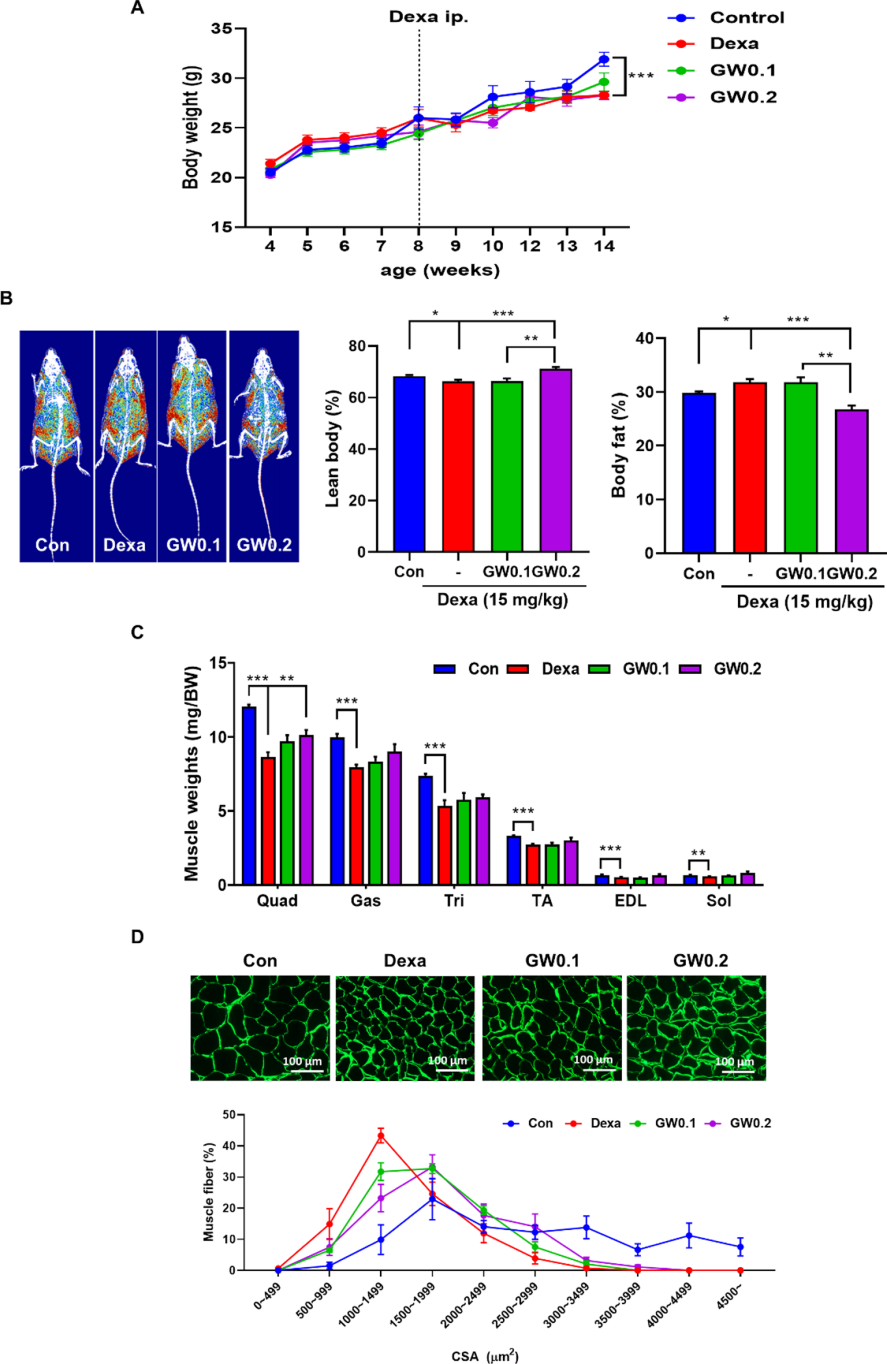
Effect of GW on muscle mass in Dexa-induced muscle atrophy. **A** Changes of body weights during the experimental period. **B** Body composition images and bar chart showing lean body mass and body fat (%). **C** The weights of skeletal muscle from the experimental mice (mg/g BW). **D** Representative image of skeletal muscle sections stained with antibody to laminin. Images were analyzed by ImageJ, and CSA were measured. Results are expressed as mean ± SEM. One-way ANOVA was used to compare more than two groups, followed by Bonferroni post-hoc test. * p < 0.05, ** p < 0.01, *** p < 0.001 versus the Dexa group

Body weight was measured weekly, and there was no significant difference in body weight among groups until 13 weeks of age. At 14 weeks, body weight was significantly lower in the Dexa group compared to the control group, and there was no significant difference in body weight among the GW0.1, GW0.2, and Dexa groups (Fig. 3A). DXA caused a decrease in lean body mass and an increase in fat mass in the Dexa group, which were reversed by GW supplementation (Fig. 3B). As there was a significant difference in lean body mass among the Dexa and GW groups, the mice were sacrificed, dissected, and their tissues were analyzed. The weights of the quadriceps (Quad), Gas, triceps (Tri), tibialis anterior (TA), extensor digitorum longus (EDL), and soleus (Sol) muscles were significantly lower in the Dexa group compared to the control group. The Quad muscle weight was significantly higher in the GW group than in the Dexa group (Fig. 3C). We measured the muscle CSA and found that the percentage of muscle fiber area was higher in the GW-supplemented group than in the Dexa group (Fig. 3D).

### GW regulates Proteostasis through the Akt/mTOR/ and GR/FoxO3a Signaling pathways

**Fig. 4:**
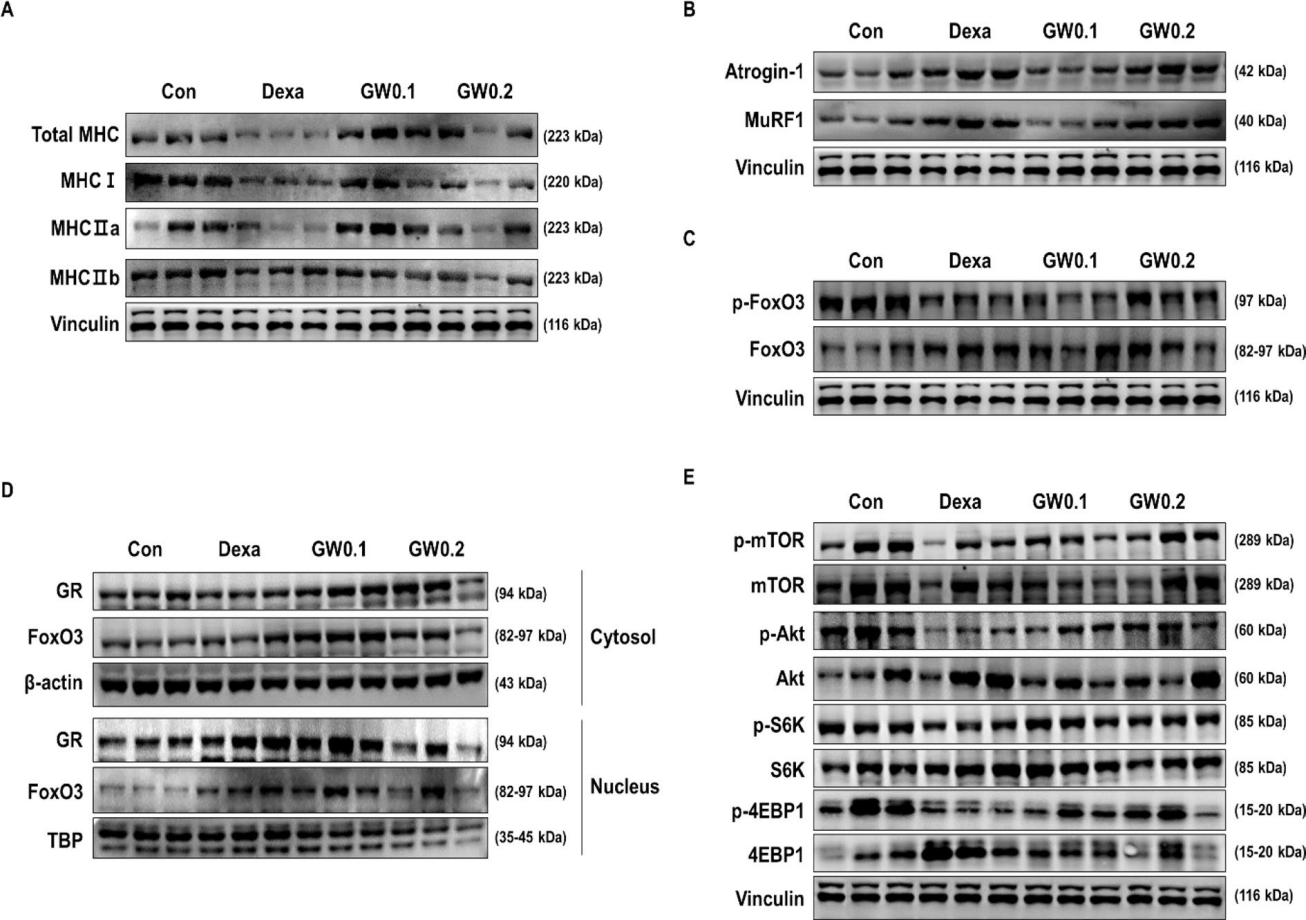
Effect of GW on muscle atrophy and protein synthesis-related factors. The protein levels of **A** total MHC, I, IIa, IIb and **B** Atrogin-1, MuRF and **C** p-FoxO3, FoxO3 in quad muscles were determined by western blot. **D** The cytosolic and nuclear expressions of GR and FoxO3a were measured. **E** The expression of p-Akt, Akt, p-mTOR, mTOR, p-S6K, S6K, p-4EBP1, 4EBP1, and Vinculin in quad muscle as determined by western blot

We performed a western blot analysis of the Quad muscle to determine the effect of the GW supplementation on muscle atrophy and protein synthesis in Dexa-induced muscle atrophy. We found a decrease in total MHC and its isomers in the Dexa group compared to the control group but an increase in the GW0.1 and GW0.2 groups compared to the Dexa group (Fig. 4A). Additionally, Dexa-induced upregulation of Atrogin-1 and MuRF1 was significantly reduced by GW supplementation (Fig. 4B). Phosphorylation of FoxO3 was decreased by Dexa treatment compared to the levels in the control group. However, GW supplementation increased the phosphorylation of FoxO3 compared to that in the Dexa group (Fig. 4C). We measured the intracellular localization of upstream regulators of atrogenes, such as GR and FoxO3a, by western blot analysis. Treatment with Dexa increased the translocation of GR and FoxO3a from the cytosol to the nucleus compared to that in the control group; however, GW led to the accumulation of GR and FoxO3 in the cytosol (Fig. 4D). Next, we examined whether GW supplementation affected protein synthesis. Phosphorylation of Akt, mTOR, S6K, and 4EBP1 was decreased by Dexa treatment compared to the levels in the control group; phosphorylation increased in the GW group compared to the levels in the Dexa group (Fig. 4E). These results suggested that GW supplementation recovered the imbalance between protein synthesis and degradation by regulating proteostasis-related signaling pathways.

### LA is the bioactive compound in GW which ameliorates muscle atrophy

**Fig. 5:**
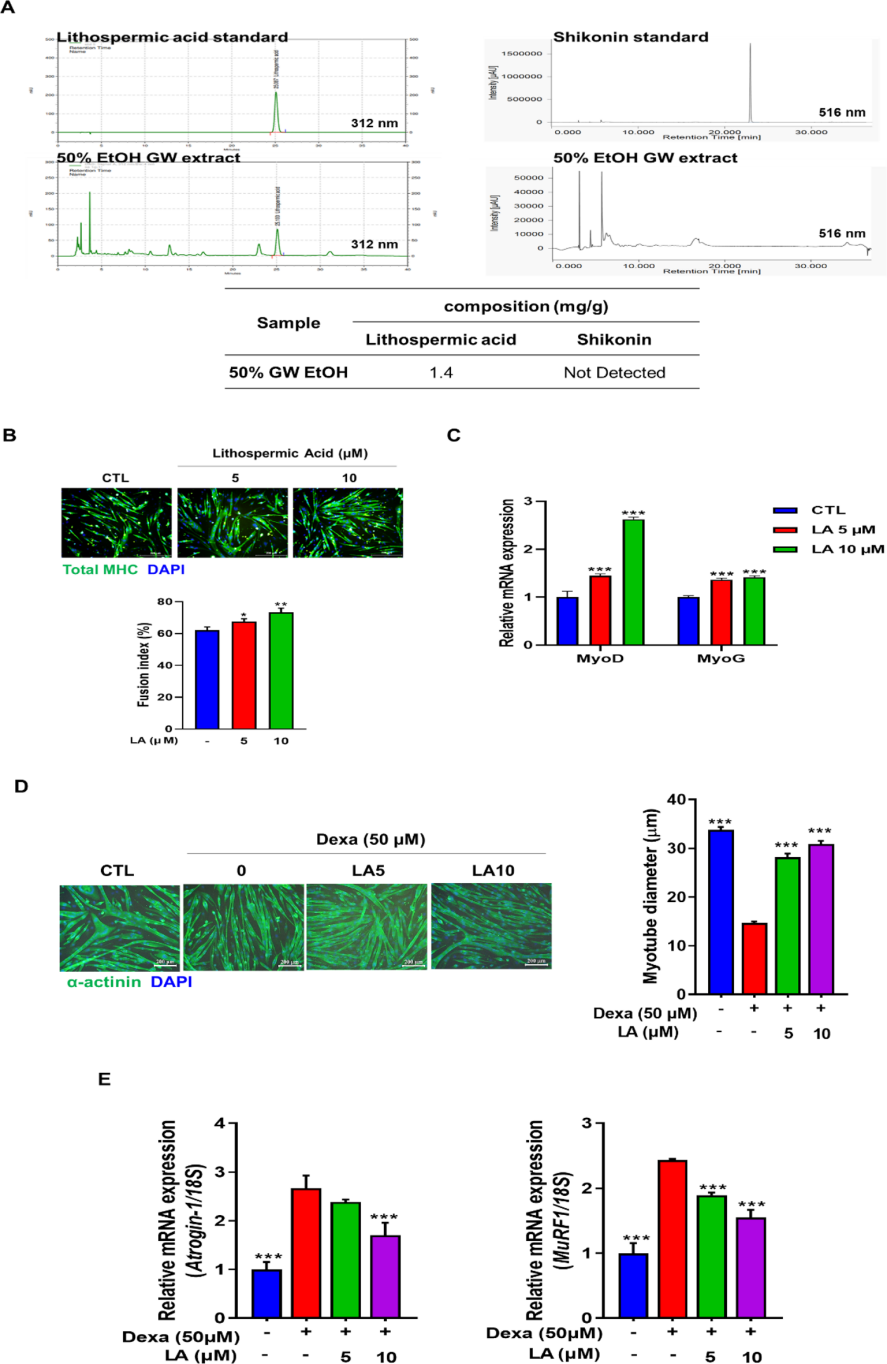
Effect of lithospermic acid on myogenesis and myotube atrophy in C2C12 cells. **A** Quantitative HPLC analysis of lithospermic acid and shikonin in GW. **B** Differentiated C2C12 cells were fixed and stained with total MHC antibody (scale bar, 200 μm). Fusion index was calculated as the average number of nuclei in MHC positive multinucleated cells above total nucleus. **C** The expression levels of MyoD and MyoG quantified by qRT-PCR in C2C12 myotubes. **D** After exposure to Dexa with/without LA, myotubes were fixed and stained with α-actinin antibody (scale bar, 200 μm). Myotube diameter was calculated as the average diameter of α-actinin-positive multinucleated myotubes. **E** The expression levels of Atrogin-1 and MuRF1 quantified by qRT-PCR in C2C12 myotubes. Results are expressed as mean ± SD. One-way ANOVA was used to compare more than two groups, followed by Bonferroni post-hoc test. * p < 0.05, ** p < 0.01, *** p < 0.001 versus the Dexa-treated myotubes

To confirm the bioactive compounds in the GW, we conducted an HPLC analysis to measure the contents of LA and shikonin. The analysis revealed an LA content of 1.4 mg/g while shikonin was undetected (Fig. 5A). C2C12 cells were treated with LA for 24 h and MTT assay was performed. We observed over 90% cell viability up to 10 µg/mL concentration (Additional file 1: Fig. S2B). We measured the effects of LA on myotube differentiation and found that LA significantly increased myogenic differentiation in a dose-dependent manner (Fig. 5B). qPCR analysis showed that myogenic regulatory factors such as MyoD and MyoG were upregulated by LA (Fig. 5C). Next, we measured the effects of LA on Dexa-induced myotube atrophy. LA significantly attenuated Dexa-induced myotube atrophy and increased myotube diameter, as determined by α-actinin IF staining (Fig. 5D). LA also effectively attenuated Dexa-induced upregulation of Atrogin-1 and MuRF1 levels in C2C12 myotubes (Fig. 5E). These results suggest that LA is the bioactive compound in GW that prevents Dexa-induced muscle atrophy.

## Discussion

Our findings showed that GW alleviated Dexa-induced muscle atrophy in C2C12 cells and mice. We demonstrated the ability of the GW to ameliorate protein degradation while enhancing protein synthesis through the Akt/mTOR/FoxO signaling pathway. Furthermore, we identified LA as the bioactive compound in the GW and suggest that it contributes to the effects exerted upon GW supplementation.

Studies on the pharmacological properties of GW have been conducted at various concentrations ranging from 100 to 500 mg/kg BW [24, 25]. We found that dietary supplementation with 0.1% (100 mg/kg BW) and 0.2% (200 mg/kg BW) GW attenuated dexamethasone-induced muscle atrophy in mice. Based on the human equivalent dose calculated according to the body surface area [26], 100 and 200 mg GW/kg in mice correspond to intakes of 0.49 and 0.97 g GW per 60 kg in humans, respectively. A previous study reported that the administration of 1.5 g GW to patients with atopic dermatitis for 10 weeks resulted in an increase in ceramide levels and improved stratum corneum hydration without any abnormalities in anthropometric measurements or blood analysis [27]. This indicates that the concentrations used in this study were within the established safety ranges.

In this study, we observed a significant decrease in body weight and lean body mass in Dexa-injected mice, along with a marked increase in body fat. Additionally, we measured the weights of various muscle types and noted significant weight loss in different muscle tissues, including the Quad, Gas, Tri, TA, EDL, and Sol. However, the GW group showed positive changes in body composition without changes in body weight or increased Quad muscle mass among the various muscle tissues in Dexa-injected mice. In skeletal muscle fibers, two primary categories prevail, slow-twitch, often referred to as type I, and fast-twitch, commonly denoted as type II [28]. Dexa-induced muscle atrophy results in a shift from fast-twitch to slow-twitch muscle fibers owing to a reduction in fast-twitch fibers [29]. In the GW group, there was an increase in Quad muscle mass, primarily composed of fast-twitch fibers and protein expression levels of MHCIIa and IIb in Dexa-injected mice. This suggests that the GW protects fast-twitch fibers from damage caused by Dexa injection.

Muscle is maintained through a balance between synthesis and breakdown. Muscle atrophy occurs when the rate of muscle wasting is higher than muscle synthesis [30]. The breakdown of muscle proteins by Atrogin-1 and MuRF1 is a major cause of muscle wasting [31], and Dexa promotes protein degradation mediated by Atrogin-1 and MuRF1, further causing muscle wasting [12, 32, 33]. As previously reported, we observed that Dexa increased the protein expressions of Atrogin-1 and MuRF1 in C2C12 cells and skeletal muscle of mice. However, administration of the GW reduced the expression of these proteins; GW supplementation decreased the protein expression of GR and FoxO3a in the nuclei of Dexa-injected mice. Considering that FoxO3 promotes the expression of Atrogin-1 and MuRF1 and activates the UPS by enhancing transcriptional activity in the nucleus [34], we speculated that the downregulation of Atrogin-1 and MuRF1 by GW may be due to the inhibition of the activity and expression of FoxO3a. We also observed that GW upregulated the phosphorylation of Akt and mTOR in Dexa-injected mice. Akt phosphorylates FoxO proteins, leading to their sequestration in the cytosol and preventing their access to target genes [35]. The Akt/mTOR signaling pathway plays a crucial role in protein synthesis at both the transcriptional and translational levels and is recognized for its ability to prevent muscle atrophy in vivo [36]. Therefore, we suggest that the Akt/mTOR/FoxO signaling pathway may contribute to increasing muscle mass and lowering the rate of muscle wasting by increasing the rate of muscle synthesis following GW supplementation.

HPLC analysis revealed that the GW contained a significant amount of LA. Shikonin, a major pigment component widely recognized as an indicator of the GW, was not detected. Previous research has shown that shikonin and its derivatives are pigmented compounds that are unstable when exposed to temperatures above 80 °C. Additionally, these substances are typically identified using n-hexane, a solvent with a lower polarity than others [37]. Therefore, we confirmed that LA is a bioactive compound in the GW. Moreover, our investigation demonstrated that LA treatment resulted in increased myotube diameter and a reduction in atrophy markers in Dexa-induced C2C12 cells. LA is a phenolic acid compound, a conjugate of rosmarinic and caffeic acids with a dihydrobenzofuran nucleus, and possesses a wide range of pharmacological properties, such as antioxidant, atherosclerosis reduction, anti-inflammatory, and anti-viral [38]. However, no studies have definitively established its effectiveness in alleviating Dexa-induced muscle atrophy, suggesting the need for further research. Nevertheless, our findings indicate the potential of LA as a bioactive component of the GW.

## Conclusions

In conclusion, we report the effect of the GW on Dexa-induced muscle atrophy in C2C12 cells and mice. We demonstrated that the GW improved protein degradation and increased protein synthesis. The present results highlight the potential of the GW to prevent and treat Dexa-induced skeletal muscle atrophy and the contribution of LA to the effects of the GW.

### Supplementary Information

**Additional file 1: Table S1.** Antibody for western blot. **Figure S1.** Comparative effect of 50% and 80% ethanol GW extract on Dexa-induced muscle atrophy in C2C12 cells. **Figure S2.** Cytotoxic effect of GW and lithospermic acid on C2C12 cells.

## Data Availability

The datasets used and/or analyzed during the current study are available from the corresponding author on reasonable request.
