# Supplementary material for: Gromwell ameliorates glucocorticoid-induced muscle atrophy through the regulation of Akt/mTOR pathway
Source: Chin Med. 2024 Jan 29;19:20. doi: 10.1186/s13020-024-00890-5 (PMC10826094; doi:10.1186/s13020-024-00890-5)
Supplement: Supplementary file 1 — Additional file 1: Table S1. Antibody for western blot. Figure S1. Comparative effect of 50% and 80% ethanol GW extract on Dexa-induced muscle atrophy in C2C12 cells. Figure S2. Cytotoxic effect of GW and lithospermic acid on C2C12 cells. [file 13020_2024_890_MOESM1_ESM.docx]

**Additional file**

**Additional Table 1. Antibody for western blot**

| Antisera | Source | | Dilution |
| --- | --- | --- | --- |
| Total MHC | DSHB | | 1:1000 |
| MHCⅠ | DSHB | | 1:1000 |
| MHCⅡa | DSHB | | 1:1000 |
| MHCⅡb | DSHB | | 1:1000 |
| Atrogin-1 (Fbx32) | Abcam | | 1:1000 |
| MuRF1 | Abcam | | 1:1000 |
| Glucocorticoid Receptor | Cell Signaling | | 1:1000 |
| FoxO3a | Cell Signaling | | 1:1000 |
| β-actin | Santa Cruz | | 1:2000 |
| TBP | Cell Signaling | | 1:2000 |
| Phospho-mTOR (Ser2448) | Cell Signaling | | 1:1000 |
| mTOR | Cell Signaling | | 1:1000 |
| Phospho-Akt (Ser473) | Cell Signaling | | 1:1000 |
| Akt | Cell Signaling | | 1:1000 |
| Phospho-p70 S6 Kinase (Thr389) | Cell Signaling | | 1:1000 |
| p70 S6 Kinase | Abcam | | 1:1000 |
| Phospho-4E-BP1 (Thr37/46) | Cell Signaling | | 1:1000 |
| 4E-BP1 | Cell Signaling | | 1:1000 |
| Vinculin | Sigma-Aldrich | 1:2000 | |


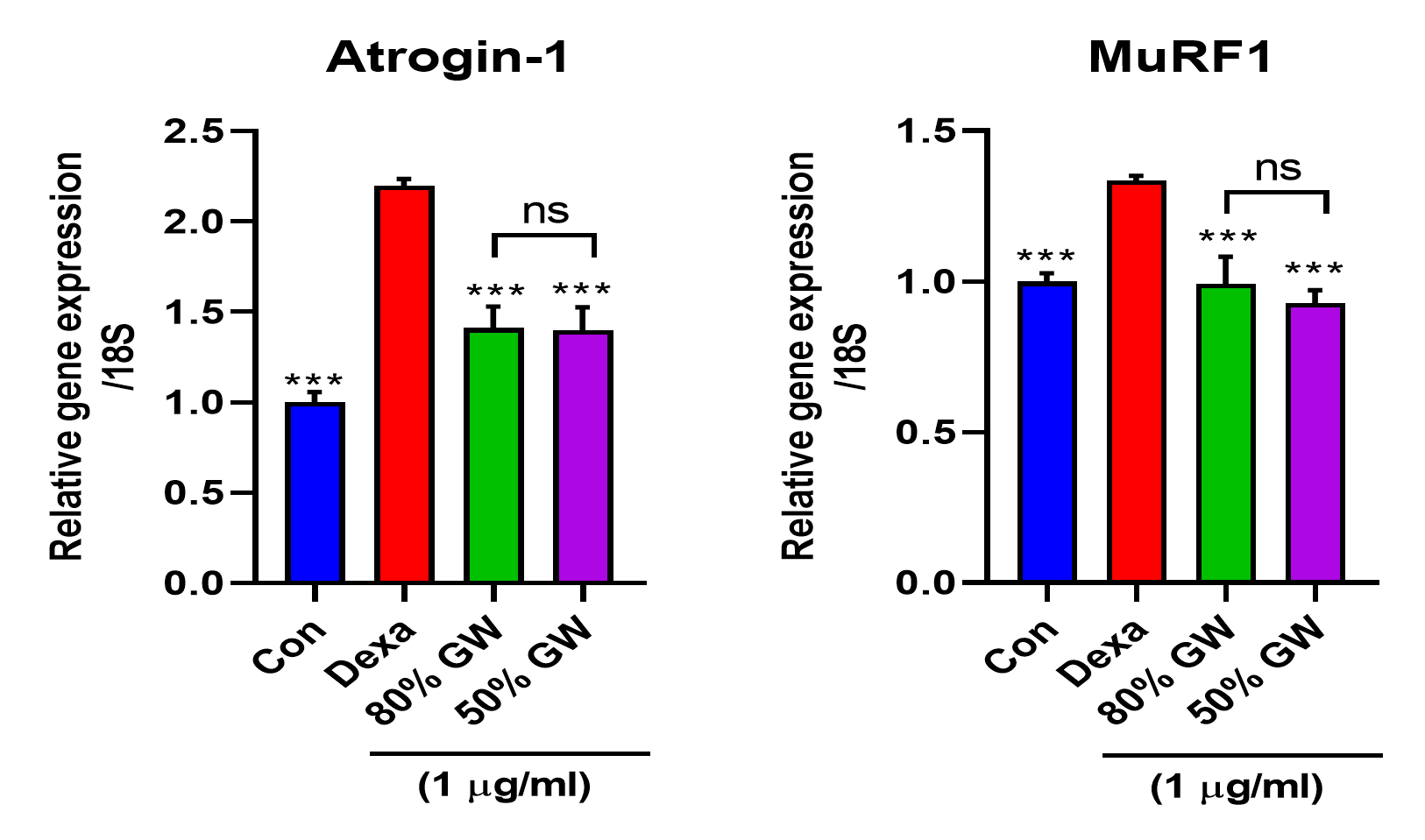


**Additional Figure 1: Comparative effect of 50% and 80% ethanol GW extract on Dexa-induced muscle atrophy in C2C12 cells**

The expression levels of Atrogin-1 and MuRF1 quantified by qRT-PCR in Dexa-treated myotubes. Results are expressed as mean ± SD. One-way ANOVA was used to compare more than two groups, followed by Bonferroni post-hoc test. * p < 0.05, ** p < 0.01, *** p < 0.001 versus the Dexa-treated myotubes.

**
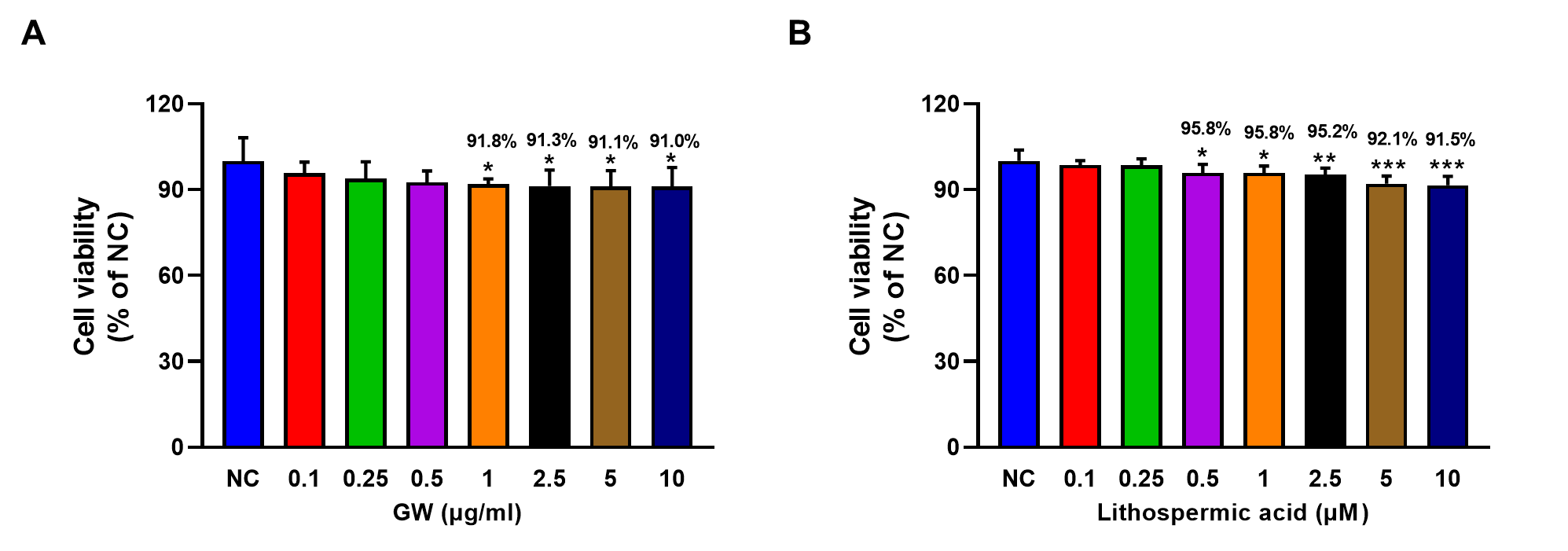
**

**Additional Figure 2: Cytotoxic effect of GW and lithospermic acid on C2C12 cells**

Cell viability of **A** GW and **B** lithospermic acid. Results are expressed as mean ± SD. One-way ANOVA was used to compare more than two groups, followed by Bonferroni post-hoc test. ** p* < 0.05, *** p* < 0.01, **** p* < 0.001 versus the NC cells.

**Measurement of cell viability**

The cell viability effect of GW and LA treatment was detected using the MTT (3-(4, 5)-dimethylthiazol−2-y1)-2, 5-diphenyltetrazolium bromide) assay. C2C12 cells were seeded at a density of 2 × 10^4^ cells/well in a 96-well plate and incubated for 24 h at 37 °C with 5% CO_2_. Then, the cells were treated with GW (0.1, 0.25, 0.5, 1, 2.5, 5, and 10 μg/ml) and LA (0.1, 0.25, 0.5, 1, 2.5, 5, and 10 μM) for an additional 24 h. To measure cell viability, 20 μL of 5 mg/mL MTT solution was added to each well and the cells were incubated for 4 h at 37 °C (in the dark). The supernatants solution was removed from all wells, and 200 μL DMSO was added to each well to dissolve the formazan crystals. Absorbance was measured at 570 nm using a Tecan microplate reader (Tecan, Mannedorf, Switzerland).
